# Study on the structure of root nodules of *Hedysarum polybotrys* Hand.-Mazz. and the isolation and identification of rhizobia

**DOI:** 10.5511/plantbiotechnology.25.0506a

**Published:** 2025-12-25

**Authors:** Tingting Liang, Xueyan Tan, Guangmao Zhang, Xinrong Li, Zhengze Qiang, Kairun Fu, Xudong Luo, Chengyi Li

**Affiliations:** 1 School of Pharmacy, Gansu University of Chinese Medicine, Lanzhou 730000, China

**Keywords:** *Hedysarum polybotrys*, nodule structure, promote growth, rhizobium, root nodules

## Abstract

Hedysari Radix, a significant Chinese herbal medicine from Northwest China’s arid region, is renowned for its unique tonic effects in traditional Chinese medicine practices. This plant, a member of the Leguminosae family, forms a symbiotic relationship with nitrogen-fixing rhizobia. However, the *Hedysarum polybotrys*-rhizobium symbiotic system remains underexplored. The root nodule structure of *H. polybotrys* was examined using an optical microscope (OM). This examination revealed that its root nodules consist of meristematic zone, infection zone, nitrogen fixation zone, and senescence zone, arranged from top to bottom. This structure suggests that the root nodules of *H. polybotrys* belong to the indeterminate nodule category. In the fields of transmission electron microscopy (TEM) and fields emission scanning electron microscopy (FESEM), significant differences were observed between infected and un-infected cells. Rhizobium, identified via 16S rRNA technology and classified as the genus *Mesorhizobium* through phylogenetic analysis. Reinoculation of rhizobium into *H. polybotrys* seedlings resulted in nodule formation on the roots. Notably, inoculated plants exhibited a considerable increase in nodule number, leaf count, leaf length, aboveground height, aboveground fresh weight, root length, and root diameter compared to uninoculated controls, demonstrating that rhizobium inoculation enhances plant growth.

## Introduction

Hedysari Radix, the dried root of *Hedysarum polybotrys* Hand.-Mazz. from the Leguminosae family, is a distinctive medicinal material from Gansu Province. Renowned as ‘Micang Hedysari Radix’, *H. polybotrys* produced in Wudu is acclaimed for its unique tonic effect, extensively utilized in traditional Chinese medicine practices. *H. polybotrys* was first documented in the Collective Notes to Canon of Materia Medica during the Liang Dynasty, noted for its capabilities in invigorating qi (Li et al. 2023) and ascending yang, consolidating the exterior and alleviating sweating, promoting diuresis and detumescence, enhancing fluid production, and nourishing blood. Pharmacological research has revealed that *H. polybotrys* possesses anti-tumor, anti-oxidant, anti-inflammatory, immune-modulating, hypoglycemic, lipid-lowering, and antihypertensive properties (Mo et al. 2022; Tsai et al. 2022; Wei et al. 2012). In traditional Chinese medicine, *H. polybotrys* is often used concurrently with Radix Astragali as qi-tonifying agents. Additionally, *H. polybotrys* enjoys popularity in regions like Taiwan, Hong Kong, Macao, and Southeast Asia for its medicinal use.

Rhizobium, a Gram-negative bacterium, forms a symbiotic nitrogen fixation system with legumes, delivering nitrogen fixation products to host plants through nodules, thus providing essential nitrogen fertilizer and other nutrients. Leguminosae medicinal plants, including commonly used species such as Astragali Radix (Yan et al. 2016, 2017), Glycyrrhizae Radix Et Rhizoma (Kusaba et al. 2021), and Sophorae Flavescentis Radix (Jiao et al. 2015; Liu et al. 2018), have identified symbiotic bacterial strains that, upon inoculation, promote growth and increase active ingredient accumulation. Despite numerous studies on *H. polybotrys*’s chemical composition (Wang et al. 2020), quality evaluation, and pharmacological effects, research on the *H. polybotrys*-rhizobium symbiotic system remains underdeveloped. Additionally, under the Chinese Pharmacopoeia’s ‘one medicine, one name’ policy, *H. polybotrys* is differentiated from Radix Astragali, impacting its cultivation and application. Products like *H. polybotrys* Oral Liquid and Fuzheng Jiedu Granules have been developed, fostering industries such as *H. polybotrys* planting and processing. However, limited research and development, small-scale industrialization, and weak application of these products have prevented *H. polybotrys* from becoming a key industry for local drug farmers to alleviate poverty and drive economic growth. Therefore, studying the *H. polybotrys*-rhizobium symbiotic system holds significant practical value for enhancing *H. polybotrys*’s quality, efficiency and local economic benefits.

This paper examines the histological characteristics and structure of *H. polybotrys* root nodules, employing paraffin sectioning, scanning electron microscopy, and transmission electron microscopy. Bacteria isolated from these nodules were identified using 16S rRNA technology. The identified rhizobia were re-inoculated to investigate the symbiotic system between *H. polybotrys* and rhizobia and to verify the growth-promoting effects on *H. polybotrys*.

## Materials and methods

### Experimental materials

*H. polybotrys* root nodules samples were collected from Zengjia Mountain (Gansu, China). The climate conditions in the area are as follows: the annual average temperature is 14.5°C, the annual precipitation is 508 mm, and the frost-free period is 239 days. *H. polybotrys* seeds were purchased from Longxi Herborist Golden Seed Co., Ltd. All reagents or chemical reagents were from commercial companies and could be used directly without processing.

### Medium and reagents

LB culture medium (Yamamoto et al. 2021): Peptone 10 g; NaCl 5 g; Yeast extract 5 g; Agar 18 g; Total volume 1,000 ml; pH 7.0. Used for pre cultivation of bacterial strains.

YMA culture medium: Mannitol 10 g; Yeast extract 0.5 g; K_2_HPO_4_ 0.3 g; MgSO_4_ 0.2 g; NaCl 0.1 g; Agar 18 g; Total volume 1,000 ml; pH 7.0. Used for isolation and purification of rhizobia.

Congo Red Improved YMA culture medium: Add 10 ml of filtered and sterilized 0.25% Congo Red solution to 1 l of sterilized YMA medium. Used for screening of rhizobia.

Ashby culture medium : K_2_HPO_4_ 0.2 g; MgSO_4_·7H_2_O 0.2 g; CaSO_4_·2H_2_O 0.1 g; NaCl 0.2 g; CaCO_3_ 5 g; Mannitol 10 g; Agar 18 g; Total volume 1,000 ml; pH 6.9. Used for identification of nitrogen fixing ability.

### Observation of nodule structure

Paraffin Section Preparation: Fresh *H. polybotrys* root nodules were initially fixed in 50% Formalin-Aceto-Alcohol (FAA) for 24 h. Subsequently, the samples underwent dehydration in a sequential alcohol series: 75% alcohol for 4 h, 85% alcohol for 2 h, 90% alcohol for 2 h, 95% alcohol for 1 h, and anhydrous ethanol twice (30 min each time). This was followed by alcohol benzene treatment for 5–10 min, and xylene immersion twice (5–10 min each time). The samples were then embedded in 65°C melting paraffin, processed thrice for 1 h each. The wax-soaked tissue was embedded and cooled on a −20°C freezing table, and the wax block was subsequently trimmed. Sections of 4 µm thickness were sliced using a paraffin slicer, flattened on a 40°C warm water spreader, and placed on a slide. The water was dried in a 60°C oven, and the tissue was then stored at room temperature.

Scanning Electron Microscopy Sample Preparation: Tissue blocks, smaller than 3 mm^2^, were rinsed with Phosphate Buffer Saline (PBS). The samples were fixed in an electron microscope fixative at room temperature for 2 h and then at 4°C. They were rinsed three times with 0.1 M phosphate buffer PB (pH 7.4), 15 min each time. The tissue was then fixed with 1% osmic acid at room temperature in the dark for 1–2 h and rinsed again three times with 0.1 M phosphate buffer PB (pH 7.4), 15 min each. The dehydration process involved a graded series of alcohol: 30%, 50%, 70%, 80%, 95%, 100%, and 100% alcohol, 15 min each step, followed by isoamyl acetate for 15 min. After drying in a critical point dryer, the samples were coated with gold using an ion sputtering instrument and subsequently observed with a scanning electron microscope.

Transmission Electron Microscopy Sample Preparation (Wang et al. 2016): The nodules were initially cut into tissue blocks of 1 mm^3^ and placed in an electron microscope fixative at room temperature for 2 h, followed by fixation at 4°C. The tissue was fixed with 1% osmic acid in 0.1 M phosphate buffer PB (pH 7.4) at room temperature in the dark for 7 h and rinsed three times with 0.1 M phosphate buffer PB (pH 7.4), 15 min each. The dehydration process involved a graded series of alcohol: 30%, 50%, 70%, 80%, 95%, 100%, and 100% alcohol, 1 h for each step. The dehydration steps involved anhydrous ethanol and acetone in a volume ratio of 3 : 1, 1 : 1, and 1 : 3, each for 0.5 h, followed by acetone for 1 h. Subsequently, the samples were infiltrated with a mixture of acetone and 812 embedding agent at 37°C in the following ratios and durations: 3 : 1 for 2–4 h, 1 : 1 overnight, and 1 : 3 for 2–4 h, before embedding in 812 embedding agent at 37°C for 5–8 h. The resin blocks were then polymerized in a 60°C oven for 48 h. The ultrathin sections of the resin blocks, measuring 60–80 nm, were prepared for staining. These sections were stained with a 2% uranyl acetate saturated alcohol solution in the dark for 8 min and washed three times with 70% alcohol and ultrapure water. This was followed by staining with a 2.6% lead citrate solution in the dark for 8 min and three washes with ultrapure water. The samples were then ready for observation under a transmission electron microscope.

### Isolation and purification of rhizobia from *H. polybotrys*

To isolate rhizobia from *H. polybotrys*, large and full nodules were processed using the plant tissue homogenate method. Initially, plant nodules were washed with PBS to eliminate impurities. Surface sterilization was carried out sequentially with 75% ethanol for 1 min, 4% NaClO for 1 min, and again with 75% ethanol for 1 min. The chopped plant root nodules were then transferred to a sterile mortar. An equal amount of normal saline was added, and the mixture was ground into a gray-white homogenate, which was then diluted 1,000 times into a sterile EP tube for use.

The diluted sample was spread continuously on the surface of a Congo red-improved YMA solid medium plate. The plate was incubated at 28°C for 5–7 days to observe bacterial growth and staining. Unstained milky white, sticky colonies were selected and streaked multiple times on YMA medium to obtain single colonies. Perform Gram staining on the bacteria using a Gram staining kit, and observe the bacterial morphology under a microscope with an oil immersion lens. The isolated and purified bacteria’s ability to produce indole acetic acid (IAA) and fix nitrogen was assessed to determine their plant growth-promotion capability. The IAA growth-promotion ability was identified following the method of Glickman and Dessaux (1995), and the bacteria’s nitrogen fixation ability was detected using the Ashby plate method (Aeron et al. 2015). The purified bacteria were preserved in a 50% glycerol solution at −80°C. Subsequent sequencing analysis was conducted to identify the bacterial strain.

### Extraction and analysis of the 16S rRNA gene sequence of the strain

The genomic DNA of the strain was extracted using a specified DNA extraction kit (Sangon Biotech (Shanghai) Co., Ltd.). The complete sequence of the 16S rRNA gene was successfully amplified through PCR. The amplified product was analyzed through 1% agarose gel electrophoresis with a voltage of 150 V and current of 100 mA.

PCR reaction composition: PCR Mix 12.5 µl; Primer F (10 µM) 1 µl; Primer R (10 µM) 1 µl; Template (DNA) 1 µl; ddH_2_O 9.5 µl. Primer F (5′-AGTTTGATCMTGGCTCAG-3′), Primer R (5′-GGTTACCTTGTTACGACTT-3′).

PCR reaction temperature conditions: Denaturation at 95°C for 5 min, followed by 30 cycles (94°C 30 s, 57°C 30 s, 72°C 90 s), and finally extended at 72°C for 10 min.

### Reinoculation experiment of rhizobium

Rhizobium cultures were grown in YMA liquid medium at 28°C and 120 r·min^−1^, and diluted bacterial liquid which the optical density at 600 nm (OD_600_) reached 0.5. The vermiculite was sterilized in an autoclave at 121°C for 20 min. Flowerpots (50×20×15 cm) were sterilized using ultraviolet light for 30 min. A low nitrogen culture solution was prepared. Preferred *H. polybotrys* seeds were disinfected with 0.1% HgCl_2_ for 5 min, followed by rinsing with sterile water five times. They were then disinfected with a 2% NaClO solution for 10 min and rinsed eight times with sterile water. The seeds were placed in a sterile petri dish (with two layers of sterile filter paper) and germinated in a solar incubator (temperature 20°C, humidity 60%, with a 12 h light/dark cycle) for 10 days before transplanting. Upon the appearance of true leaves, 1 ml of the bacterial solution was injected into the roots for inoculation, with a supplement added on the seventh day. Observations were conducted 90 days post-inoculation. The nitrogenase activity in root nodules was determined using a nitrogenase enzyme-linked immunosorbent assay (ELISA) kit.

## Results

### Observation of nodule structure

**Figure 1:**
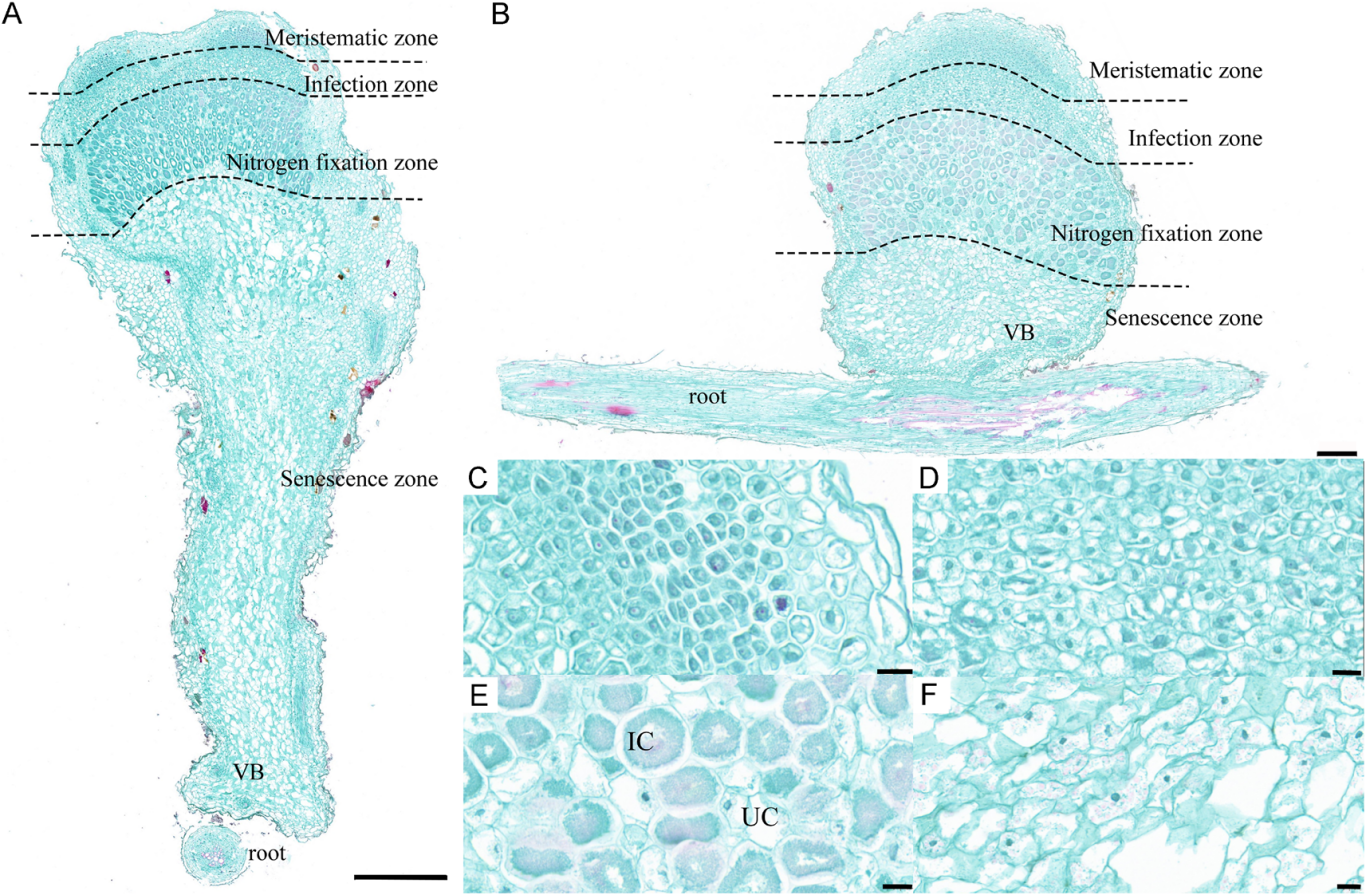
Figure 1. The histological structure of *H. polybotrys* root nodules. (A–B) Root nodule longitudinal section; (C) The cells in meristematic zone; (D) The cells in infection zone; (E) The cells in nitrogen fixation zone; (F) The cells in senescence zone. IC, infected cell; UC, un-infected cell; VB, vascular bundle. Scale bars, 500 µm in (A), 200 µm in (B), 20 µm in (C–F).

Microscopic examination of paraffin sections (Figure 1A, B) revealed the structural composition of *H. polybotrys* root nodules. These nodules comprise meristematic zone, infection zone, nitrogen fixation zone, and senescence zone, arranged from the top to the bottom. The meristematic zone cells (Figure 1C) are small, with diameters ranging from 8 µm to 13 µm, featuring a long-axis and equal-diameter shape. These cells possess a prominent, neatly arranged, dense nucleus with minimal intercellular space. In the infected area (Figure 1D), cells are slightly larger, measuring between 11 µm and 26 µm in diameter, and exhibit round to oval shapes with close arrangement. The nitrogen fixation area cells (Figure 1E) are notably larger, with diameters of 25 µm to 45 µm. These mostly oval cells are densely packed with bacteroids, filling the entire infected cell, with a few cells remaining uninfected. In the senescent area (Figure 1F), the cells reduce in size, ranging from 29 µm to 39 µm in diameter, displaying irregular shapes and signs of disintegration.

**Figure 2:**
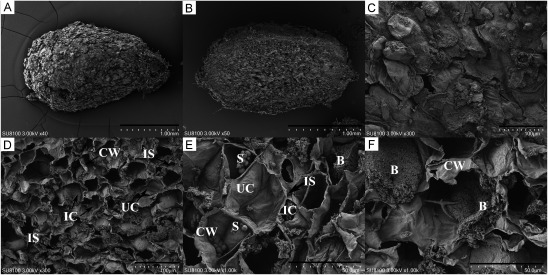
Figure 2. Observation of *H. polybotrys* root nodules by scanning electron microscope. (A, C) The surface of root nodules; (B, D–F) Root nodule longitudinal section. B, bacteroid; CW, cell wall; IC, infected cell; IS, intercellular space; S, starch grain; UC, un-infected cell. Scale bars, 1 mm in (A–B), 100 µm in (C–D), 50 µm in (E–F).

Scanning electron microscope images show that the surface of *H. polybotrys* root nodules (Figure 2A, C) is uneven, characterized by numerous folds and some breakage. There is a clear division between the non-infected and infected areas (Figure 2D–E). The most significant difference between un-infected and infected cells is the absence of rhizobia in the former. The contents of the un-infected cells appear granular or agglomerated, unevenly distributed, with spherical starch granules visible. The infected cells (Figure 2F), however, are turgid, containing numerous long, rod-shaped rhizobium bacteria, with discernible cell walls and intercellular spaces.

**Figure 3:**
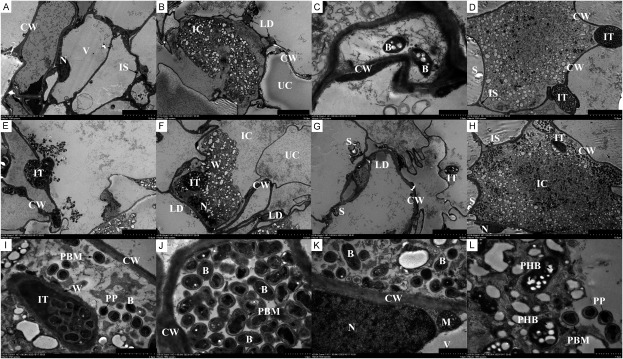
Figure 3. Observation of *H. polybotrys* root nodules by transmission electron microscopy. (A–L) Root nodule cells have organelles such as nucleus and mitochondria. Infected cells contain a large number of bacteroids, and infection lines can be clearly seen. B, bacteroid; CW, cell wall; IC, infected cell; IS, intercellular space; IT, infection thread; M, mitochondria; N, nucleus; LD, lipid droplets; PBM, peribacteroid membrane; S, starch grain; W, infected line wall; UC, un-infected cell; V, vacuole; PP, polyphosphate particle; PHB, poly-β-hydroxybutyrate. Scale bars, 10 µm in (A–B), 1 µm in (C, J–L), 10 µm in (D–H), 2 µm in (I).

Transmission electron microscopy revealed significant differences in the composition of infected and un-infected cells within Hedysari root nodules. The un-infected cells (Figure 3A) exhibited high vacuolization, with organelles compressed around the cell wall. These cells had uneven cell wall thickness and their vacuoles contained a small quantity of free fibrous or granular substances. There are infection lines in some nodule cells, through which rhizobia infect cells. The infection line releases rhizobia to complete the infection process (Figure 3C, D, E). The nucleus (Figure 3F) and mitochondria (Figure 3K) were visible in the infected cells. Due to the increased number of rhizobia, the organelles, such as mitochondria in the infected cells, were pushed to the periphery of the cell wall (Figure 3F).

Rhizobium differentiates into bacteroids after invading root nodule cells. These infected cells contain numerous bacteroids of varying lengths and uneven thickness, displaying a diversity of shapes like rod-shaped, pear-shaped, oval-shaped, and dumbbell-shaped (Figure 3J–L). When the volume of bacteroid is small, the bacteroid is filled with small black round particles, identified as polyphosphate (PP) particles. These PP particles provide essential substances for the synthesis of amino acids, bases, sugars, fats, and other vital substances for immature bacteria. In contrast, when the volume of bacteroid is large, numerous white spots with high electron density appear, identified as poly-β-hydroxybutyrate (PHB), a natural product involved in cell metabolism as a nutrient and energy storage substance.

### Isolation and purification of rhizobia from *H. polybotrys*

The rhizobia isolated from *H. polybotrys* exhibited robust growth on YMA medium. Examination of colony morphology revealed that the single colonies of the strain were generally round, wet, translucent, milky white, and slightly convex (Supplementary Figure S1A–B). After Gram staining, Gram-negative bacilli were observed under a microscope (Supplementary Figure S1C). In tests for IAA production and nitrogen fixation, the bacterial solution turned significantly red after Salkowski’s colorimetric reaction, indicating IAA production capability (Supplementary Figure S1D). The formation of a large, transparent nitrogen fixation ring in Ashby nitrogen-free medium indicated good growth and strong nitrogen fixation ability (Supplementary Figure S1E).

### Analysis of 16S rRNA gene sequence of strains

Bacterial DNA was extracted from 5 nodules and 16S rRNA gene was amplified by PCR, and then sent to a sequencing company for analysis. Homologous search analysis was conducted in the NCBI database, comparing the gene similarity with ten similar model strains. Based on the similarity of its gene sequence, the strain was identified as belonging to the genus *Mesorhizobium*. A comparative analysis of gene similarity revealed a 100% match with *Mesorhizobium amorphae* CCNWGS0123 (CP015318.1) (Supplementary Table S1).

**Figure 4:**
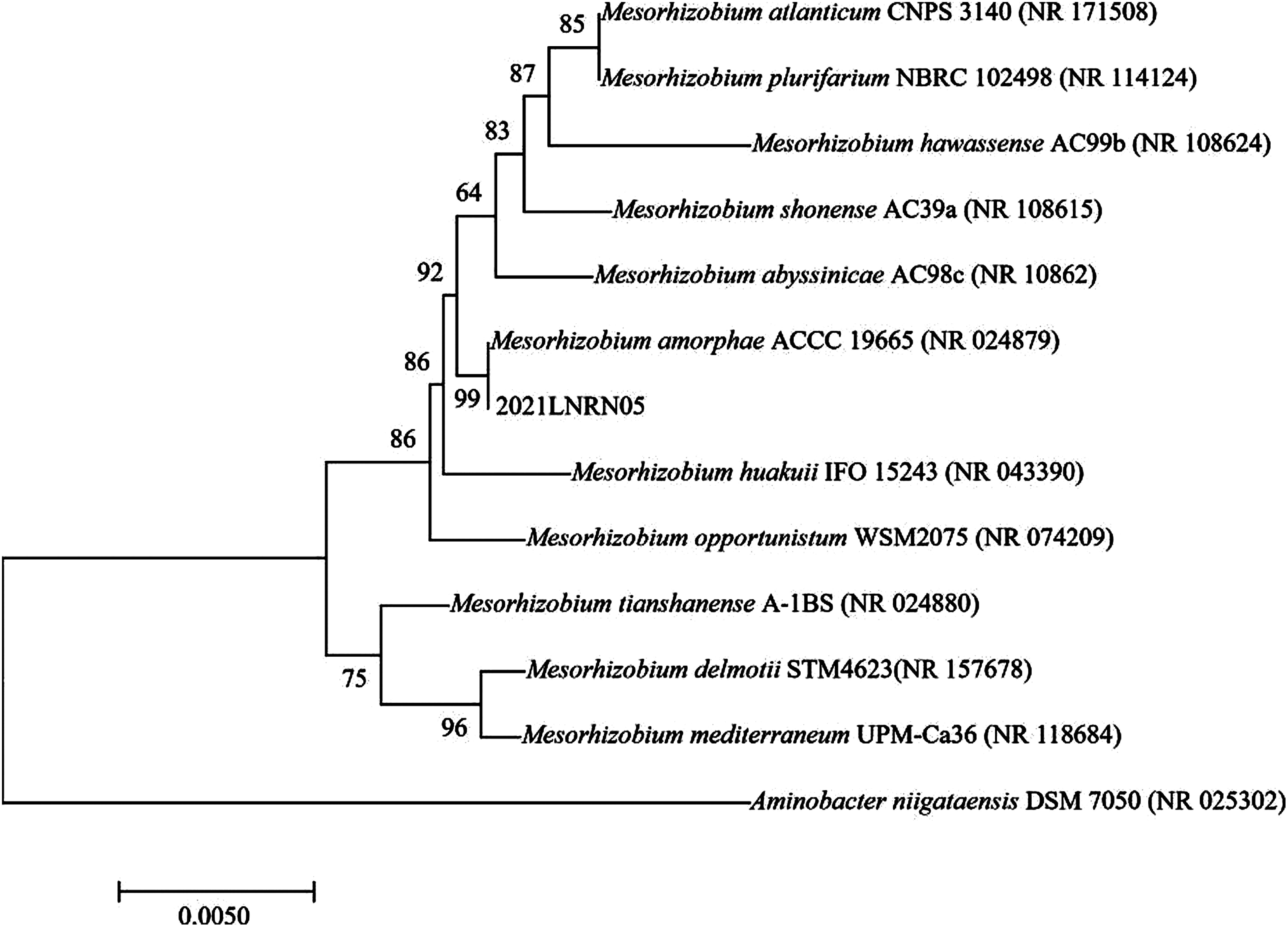
Figure 4. Phylogenetic tree based on 16S rRNA gene sequences.

Through PUBMED BLAST website analysis and comparison, it was found that the isolate 2021LNRN05 had the highest homology with *Mesorhizobium amorphae*. The phylogenetic tree is shown in Figure 4. MEGA 7.0 was used to construct the phylogenetic tree, and it was found that it had the closest genetic relationship with *Mesorhizobium amorphae*.

### Reinoculation experiment of rhizobium

**Figure 5:**
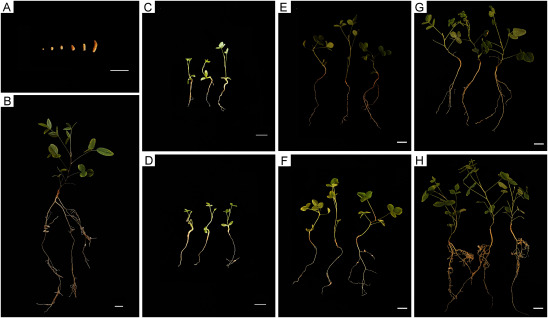
Figure 5. Growth process diagram of *H. polybotrys* after rhizobia inoculation. (A) Nodule morphology at different stages; (B) 90 d experimental group picture. (C–D) 30 d observation picture; (E–F) 60 d observation picture; (G–H) 90 d observation picture. (C, E, G) Blank group picture. (D, F, H) Experimental group picture. Scale bars, 1 cm in (A–B) and (E–H), 0.5 cm in (C–D).

Continuous observation of *H. polybotrys* seedlings after rhizobium reinoculation revealed notable changes in the roots. Nodules began appearing 25 days post-inoculation, evolving in shape from round to oval, elongated, and irregularly oval, and changing in color from yellow-white to yellow-brown. Root nodules were predominantly found in the fibrous roots (Figure 5B). Thirty days after rhizobium inoculation (Figure 5D), nodulation commenced in the plants of the experimental group. At this stage, there was no significant difference in the growth indices of the above-ground parts and root length compared to the control group. Sixty days post-inoculation (Figure 5F), an increase in the number of nodules was observed in the experimental group, along with noticeable enhancements in leaf size and root length. Ninety days after inoculation (Figure 5B, H), all plants in the experimental group exhibited nodulation, with a significant increase in the number of leaves, root length, and fibrous roots, surpassing the control group. The nitrogenase activity in root nodules was 38.28±0.70 U/g, demonstrated strong nitrogen-fixing activity.

**Figure 6:**
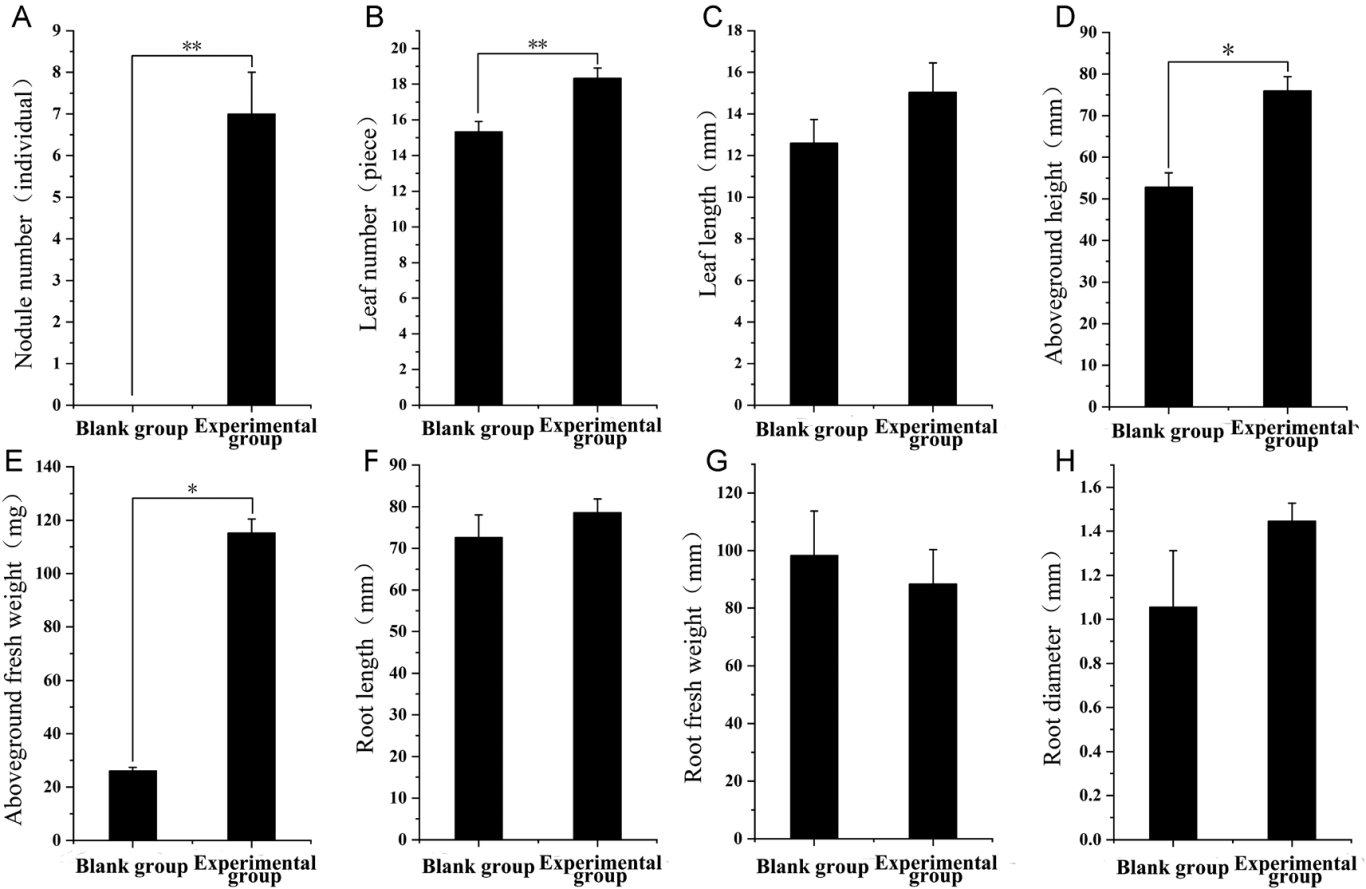
Figure 6. Comparison of the growth and symbiosis parameters of inoculated and uninoculated *H. polybotrys* plants at 90 days after inoculation (*n*=3). (A) Nodule number; (B) Leaf number; (C) Leaf length; (D) Aboveground height; (E) Aboveground fresh weight; (F) Root length; (G) Root fresh weight; (H) Root diameter.

There were 3 samples measured each group. At this point, the experimental group showed higher values in several metrics compared to the blank group (Figure 6): leaf number by 20.0%, leaf length by 19.4%, aboveground height by 43.7%, aboveground fresh weight by 341.5%, root length by 8.2%, root diameter by 36.9%, and a decrease in root fresh weight by 10.0%.

## Discussion

The study of leguminous plant nodules reveals a division into two types: determinate nodules and indeterminate nodules (Ferguson et al. 2010). There is a noted correlation between the type of nodule, and its shape and size. Determinate nodules typically present a round shape, and their root nodule primordium is located in the outer cortex. These nodules possess an inactive meristem, which allows only limited growth (Foucher and Kondorosi 2000). In contrast, indeterminate nodules usually have an irregular shape, with the root nodule primordium located in the endodermis, retaining an active apical meristem. Furthermore, indeterminate nodules can be categorized into several sections with different age gradients from the bottom to the top, including intracellular saprophytic zone, senescence zone, nitrogen fixation zone, transition zone, infection zone, and apical meristem zone (Hirsch 1992). The results of the paraffin section analysis in this study differ from those observed in finite nodules, such as *Dalbergia odorifera*, plant soybean, and *Arachis hypogaea*. Instead, they align with the characteristics of indeterminate nodules observed in species like *Robinia pseudoacacia* and *Leucaena leucocephala*. The occurrence of multiple bifurcations and morphological diversity in the study indicates that the root nodules of *H. polybotrys* belong to the indeterminate nodule category.

Scanning electron microscopy analysis of *H. polybotrys* nodules revealed significant differences between un-infected and infected cells. Infected cells contained numerous bacteroids, indicative of nitrogen fixation metabolic activity. The presence of starch granules in nodule cells suggests a nutritional microenvironment conducive to the development of bacteroids formed by infected cells. Transmission electron microscopy identified PP and PHB particles within bacteroids. The content and distribution of these particles vary among bacteroids, potentially correlating with their developmental stages. In the early growth phase of bacteroids, PP predominates, providing a material foundation for their growth. During the middle and late growth stages, PHB plays a role in nitrogen fixation as a nutrient and energy storage substance. The specific periods and pathways of their production warrant further investigation. Within the infected cells, bacteroids are enclosed in a vesicle membrane, also known as the peribacteroid membrane. This membrane serves to protect both the bacteroid and the host plant cells, ensuring the normal transfer of materials, energy metabolism, and information exchange between them (Xing et al. 2021).

Based on the findings, a selective medium was utilized to isolate and screen the rhizobia of *H. polybotrys*. Through experiments evaluating nitrogen fixation and IAA bacterial growth-promoting abilities, it was determined that the isolated rhizobia of *H. polybotrys* possessed growth-promoting capabilities. Using 16S rRNA molecular identification technology, the rhizobium of *H. polybotrys* was identified as the genus *Mesorhizobium*. This identification aligns with other scholarly findings regarding the rhizobium of *Astragalus membranaceus* (Chen et al. 2015; Wei et al. 2003; Zhao et al. 2008), where the genus *Mesorhizobium* is the dominant symbiotic bacterium.

Auxin (IAA) is a crucial hormone for plant growth and development, regulating various biological processes like cell division, elongation, differentiation, seed germination, root hair growth, fruit development, and senescence. Over 80% of rhizosphere microorganisms, including rhizobia, can synthesize and release auxin (Alemneh et al. 2020; Singh et al. 2021). Rhizobium shows potential in promoting the growth of leguminous medicinal plants, increasing yield, enhancing plant stress resistance, and accumulating active components (Meleta and Abera 2019; Mushtaq et al. 2021; Wu et al. 2021). When rhizobia isolated from the root nodules of *H. polybotrys* were reinoculated, they significantly increased the number of leaves, height, root length, and root diameter of *H. polybotrys*, although the root weight was not notably affected. Nodule formation, a process requiring substantial energy (Magori and Kawaguchi 2009), may exert long-distance control over nodule formation, impacting root matter accumulation and growth in *H. polybotrys*. Overall, rhizobium plays a vital role in promoting the growth and development of *H. polybotrys*.

The enhancement of effective components and yield in leguminous medicinal plants is a key area of focus, with the relationship between rhizobia and these plants being integral. A thorough investigation of the *H. polybotrys*-rhizobium symbiotic system, and understanding the correlation between rhizobium and the quality of *H. polybotrys*, offers a theoretical foundation for enhancing both the yield and quality of *H. polybotrys*. In the context of promoting environmental protection and the sustainable development and conservation of traditional Chinese medicine resources, delving into the dynamics of this symbiosis will be a crucial direction for future research.

## Conclusion

This study aimed to explore the histological characteristics and structure of *H. polybotrys* root nodules, isolate and identify rhizobia strains of *H. polybotrys* root nodules, and study the role of rhizobia in promoting the growth of *H. polybotrys* root nodules. Paraffin sections of root nodules were observed using an optical microscope which showed that the nodules of *H. polybotrys* were the indeterminate nodules. The rhizobia of *H. polybotrys* belong to the genus *Mesorhizobium* via the 16S rRNA technology. Reinoculation of rhizobia significantly promoted the growth of *H. polybotrys* seedlings. The results of this study provide new basic data for the study of *H. polybotrys*-rhizobia symbiosis system, but the growth promotion mechanism needs to be studied. The next step will also be to carry out research on *H. polybotrys* biofertilizer to further improve the quality of *H. polybotrys*.

## Description of supplementary files

Supplementary Figure S1. Strain characteristics.

Supplementary Table S1. 16S rRNA gene sequence homology comparison table.

## References

[RAeron2015] Aeron A, Chauhan PS, Dubey RC, Maheshwari DK, Bajpai VK (2015) Root nodule bacteria from *Clitoria ternatea* L. are putative invasive nonrhizobial endophytes. *Can J Microbiol* 61: 131–142 25619106 10.1139/cjm-2014-0483

[RAlemneh2020] Alemneh AA, Zhou Y, Ryder MH, Denton MD (2020) Mechanisms in plant growth-promoting rhizobacteria that enhance legume-rhizobial symbioses. *J Appl Microbiol* 129: 1133–1156 32592603 10.1111/jam.14754

[RChen2015] Chen W, Sun L, Lu J, Bi L, Wang E, Wei G (2015) Diverse nodule bacteria were associated with *Astragalus* species in arid region of northwestern China. *J Basic Microbiol* 55: 121–128 24115208 10.1002/jobm.201300209

[RFerguson2010] Ferguson BJ, Indrasumunar A, Hayashi S, Lin MH, Lin YH, Reid DE, Gresshoff PM (2010) Molecular analysis of legume nodule development and autoregulation. *J Integr Plant Biol* 52: 61–76 20074141 10.1111/j.1744-7909.2010.00899.x

[RFoucher2000] Foucher F, Kondorosi E (2000) Cell cycle regulation in the course of nodule organogenesis in Medicago. *Plant Mol Biol* 43: 773–786 11089876 10.1023/a:1006405029600

[RGlickmann1995] Glickmann E, Dessaux YA (1995) A critical examination of the specificity of the salkowski reagent for indolic compounds produced by phytopathogenic bacteria. *Appl Environ Microbiol* 61: 793–796 16534942 10.1128/aem.61.2.793-796.1995 PMC1388360

[RHirsch1992] Hirsch AM (1992) Developmental biology of legume nodulation. *New Phytol* 122: 211–237 33873995 10.1111/j.1469-8137.1992.tb04227.x

[RJiao2015] Jiao YS, Liu H, Yan H, Wang E, Tian C, Chen W, Guo B, Chen W (2015) Rhizobial diversity and nodulation characteristics of the extremely promiscuous legume *Sophora flavescens.* *Mol Plant Microbe Interact* 28: 1338–1352 26389798 10.1094/MPMI-06-15-0141-R

[RKusaba2021] Kusaba I, Nakao T, Maita H, Sato S, Chijiiwa R, Yamada E, Arima S, Kojoma M, Ishimaru K, Akashi R, et al. (2021) *Mesorhizobium* sp. J8 can establish symbiosis with *Glycyrrhiza uralensis*, increasing glycyrrhizin production. *Plant Biotechnol (Tokyo)* 38: 57–66 34177325 10.5511/plantbiotechnology.20.1124a PMC8215473

[RLi2023] Li Y, Zhang Y, Cao R, Niu J, Bian T, Ma D, Wang Z, Wang M, Yan X (2023) Identifications of metabolic differences between Hedysari Radix Praeparata Cum Melle and Astragali Radix Praeparata Cum Melle for spleen-qi deficiency rats: A comparative study. *J Pharm Biomed Anal* 236: 115689 37677887 10.1016/j.jpba.2023.115689

[RLiu2018] Liu YH, Jiao Y, Liu L, Wang D, Tian C, Wang E, Wang L, Chen W, Wu S, Guo B, et al. (2018) Nonspecific symbiosis between *Sophora flavescens* and different rhizobia. *Mol Plant Microbe Interact* 31: 224–232 29173048 10.1094/MPMI-05-17-0117-R

[RMagori2009] Magori S, Kawaguchi M (2009) Long-distance control of nodulation: Molecules and models. *Mol Cells* 27: 129–134 19277493 10.1007/s10059-009-0016-0

[RMeleta2019] Meleta T, Abera G (2019) Effects of *Rhizobium* inoculation and phosphorus fertilizer rates on growth yield and yield components of chickpea (*Cicer arietinum* L.) at Goro Bale Zone Oromia Regional State. *Int J Appl Agric Sci* 5: 62–70

[RMo2022] Mo X, Guo D, Jiang Y, Chen P, Huang L (2022) Isolation, structures and bioactivities of the polysaccharides from *Radix Hedysari*: A review. *Int J Biol Macromol* 199: 212–222 34995662 10.1016/j.ijbiomac.2021.12.095

[RMushtaq2021] Mushtaq Z, Faizan S, Gulzar B, Hakeem KR (2021) Inoculation of *Rhizobium* alleviates salinity stress through modulation of growth characteristics physiological and biochemical attributes stomatal activities and antioxidant defence in *Cicer arietinum* L. *J Plant Growth Regul* 40: 2148–2163

[RSingh2021] Singh K, Gera R, Sharma R, Maithani D, Chandra D, Bhat MA, Kumar R, Bhatt P (2021) Mechanism and application of *Sesbania* root-nodulating bacteria: An alternative for chemical fertilizers and sustainable development. *Arch Microbiol* 203: 1259–1270 33388789 10.1007/s00203-020-02137-x

[RTsai2022] Tsai Y, Lin M, Peng W, Tseng C, Lee M, Yang B, Chang W (2022) Comparison of the immunomodulatory effect of TCM formulas containing either Astragali Radix or with this replaced by Hedysari Radix. *Nat Prod Commun* 17: 1–11

[RWang2020] Wang B, Liu X, Xue Z, Yang X, Fang Y, Feng S (2020) Comparative study of ultra-high-performance supercritical fluid chromatography and ultra-high-performance liquid chromatography to simultaneous determination of ten components in *Radix hedysari*. *Pharmacogn Mag* 16: 99–110

[RWang2016] Wang C, Yu H, Luo L, Duan L, Cai L, He X, Wen J, Mysore KS, Li G, Xiao A, et al. (2016) NODULES WITH ACTIVATED DEFENSE 1 is required for maintenance of rhizobial endosymbiosis in *Medicago truncatula*. *New Phytol* 212: 176–191 27245091 10.1111/nph.14017

[RWei2012] Wei D, Cheng W, Wei Y, Zhang L (2012) Phosphorylated modification and *in vitro* antioxidant activity of Radix Hedysari polysaccharide. *Glycoconj J* 29: 167–172 22535466 10.1007/s10719-012-9377-2

[RWei2003] Wei GH, Tan Z, Zhu M, Wang E, Han S, Chen W (2003) Characterization of rhizobia isolated from legume species within the genera *Astragalus* and *Lespedeza* grown in the Loess Plateau of China and description of *Rhizobium loessense* sp. nov. *Int J Syst Evol Microbiol* 53: 1575–1583 13130051 10.1099/ijs.0.02031-0

[RWu2021] Wu ZY, Meng X, Jiao Y, Guo B, Sui X, Ma S, Chen W, Singh RP (2021) *Bradyrhizobium arachidis* mediated enhancement of (oxy)matrine content in the medicinal legume *Sophora flavescens.* *Lett Appl Microbiol* 72: 570–577 33474743 10.1111/lam.13453

[RXing2021] Xing J, Zhang L, Duan Z, Lin J (2021) Coordination of phospholipid-based signaling and membrane trafficking in plant immunity. *Trends Plant Sci* 26: 407–420 33309101 10.1016/j.tplants.2020.11.010

[RYamamoto2021] Yamamoto K, Toya S, Sabidi S, Hoshiko Y, Maeda T (2021) Diluted Luria-Bertani medium vs. sewage sludge as growth media: Comparison of community structure and diversity in the culturable bacteria. *Appl Microbiol Biotechnol* 105: 3787–3798 33856534 10.1007/s00253-021-11248-4

[RYan2016] Yan H, Ji Z, Jiao Y, Wang E, Chen W, Guo B, Chen W (2016) Genetic diversity and distribution of rhizobia associated with the medicinal legumes *Astragalus* spp. and *Hedysarum polybotrys* in agricultural soils. *Syst Appl Microbiol* 39: 141–149 26915496 10.1016/j.syapm.2016.01.004

[RYan2017] Yan H, Xie J, Ji Z, Yuan N, Tian C, Ji S, Wu Z, Zhong L, Chen W, Du Z, et al. (2017) Evolutionarily conserved nodE, nodO, T1SS, and hydrogenase system in rhizobia of *Astragalus membranaceus* and *Caragana intermedia.* *Front Microbiol* 8: 2282 29209294 10.3389/fmicb.2017.02282 PMC5702008

[RZhao2008] Zhao CT, Wang E, Chen W, Chen W (2008) Diverse genomic species and evidences of symbiotic gene lateral transfer detected among the rhizobia associated with *Astragalus* species grown in the temperate regions of China. *FEMS Microbiol Lett* 286: 263–273 18657113 10.1111/j.1574-6968.2008.01282.x

