# Supplementary material for: Study on the structure of root nodules of Hedysarum polybotrys Hand.-Mazz. and the isolation and identification of rhizobia
Source: Plant Biotechnol (Tokyo). 2025 Dec 25;42(4):431–9. doi: 10.5511/plantbiotechnology.25.0506a (PMC12781901; doi:10.5511/plantbiotechnology.25.0506a)
Supplement: Supplementary Data [file plantbiotechnology-42-4-25.0506a-s001.pdf]

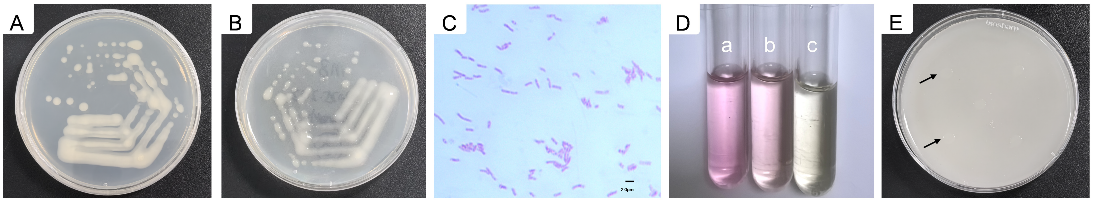

Supplementary Figure S1. Strain characteristics. (A-B) The colonies of the strain; (C) Gram stain; (D) IAA production, a: 10 μg/mL reference standard, b: Sample group, c: Blank group; (E) Nitrogen fixation. Scale bars, 20 μm in (C).

Supplementary Table S1. 16S rRNA gene sequence homology comparison table

Description	Max Score	Total Score	Query Cover	E value	Per. Ident	Accession
Mesorhizobium amorphae CCNWGS0123, complete genome	2630	5261	100%	0.0	100%	CP015318.1
Mesorhizobium sp. CCANP87 partial 16S rRNA gene, isolate CCANP87	2630	2630	100%	0.0	100%	HF931067.1
